# The Statistical Analysis of Multi-Voxel Patterns in Functional Imaging

**DOI:** 10.1371/journal.pone.0069328

**Published:** 2013-07-08

**Authors:** Kai Schreiber, Bart Krekelberg

**Affiliations:** Center for Molecular and Behavioral Neuroscience, Rutgers University, Newark, New Jersey, United States of America; University of Minnesota, United States of America

## Abstract

The goal of multi-voxel pattern analysis (MVPA) in BOLD imaging is to determine whether patterns of activation across multiple voxels change with experimental conditions. MVPA is a powerful technique, its use is rapidly growing, but it poses serious statistical challenges. For instance, it is well-known that the slow nature of the BOLD response can lead to greatly exaggerated performance estimates. Methods are available to avoid this overestimation, and we present those here in tutorial fashion. We go on to show that, even with these methods, standard tests of significance such as Students’ T and the binomial tests are invalid in typical MRI experiments. Only a carefully constructed permutation test correctly assesses statistical significance. Furthermore, our simulations show that performance estimates increase with both temporal as well as spatial signal correlations among multiple voxels. This dependence implies that a comparison of MVPA performance between areas, between subjects, or even between BOLD signals that have been preprocessed in different ways needs great care.

## Introduction

One of the strengths of functional magnetic resonance imaging (fMRI) is that it provides information on the whole brain in a single experiment. A typical experiment generates BOLD responses for around 500,000 separate small cubes (voxels) across the human brain. While most data analyses consider each of these voxels separately, a growing field of research analyzes the multivariate patterns of BOLD responses. This so-called multi-voxel pattern analysis (MVPA) has been used successfully to demonstrate the encoding of a wide range of information in the BOLD response in specific brain regions (e.g. faces and objects [1], orientation [2], and motion [3]) Although we do not agree with all of their recommendations concerning the statistical analysis of multi-voxel patterns, good introductions and overviews of the MVPA method can be found in [4–6].

In a typical MVPA study, the subject in the scanner is made to alternate between two or more states (e.g. viewing houses or faces, or feeling sad vs. happy) and the researcher investigates whether one can determine the state of the subject from the pattern of BOLD activity in a certain brain area. This is a pattern classification task: is there a pattern of BOLD activity that corresponds to ‘House’ but not ‘Face’, or ‘Sad’ and not ‘Happy’?

To analyze such data, the researcher feeds BOLD responses together with the correct class labels to a classification algorithm [2]. (For instance, the BOLD response in the voxels of the fusiform face area in 10 trials in which the subject saw a house and 10 trials in which the subject saw a face.) On the basis of this training set, the algorithm determines a classifier that best captures the pattern structure of the training set. Then, the researcher provides a new set of BOLD responses to the classifier (5 trials in which faces were shown, 5 in which houses were shown), which returns predicted class labels (‘house’, ’house’, ’face’,…) for all the trials. These predictions are compared to the correct class labels to determine the percentage of trials classified correctly. If this performance of the classifier is significantly above chance, one concludes that the BOLD responses contain information about the classified states, and infers that the brain area where the signals originated is somehow involved in the neural representation of these states.

While such an analysis sounds straightforward in theory, there are serious issues in applying this procedure to BOLD imaging data. We consider three separate problems in the three subsections of the Results. The first is estimation; the problem of obtaining an unbiased estimate of the (average) performance of the classifier. It is well known that estimation is affected by the slow temporal correlations in the BOLD signal, and multiple researchers have suggested the solution that we discuss here (Leave-Block-Out cross-validation). We present it here only for completeness and to provide context to understand the other two problems, but refer to [4] for a fuller description. The second problem is significance testing. We show that some of the existing approaches can lead to greatly inflated estimates of significance (even when the average performance has been estimated correctly). Building on previous work [7,8] we show that a carefully constructed permutation test provides a correct estimate of significance. The third problem is the comparison of performance estimates. Our simulations illustrate the influence of spatial and temporal correlations on classification performance. Given that spatial and temporal correlations could be introduced by differences in hemodynamic coupling across regions or subject groups, or even by differences in data preprocessing, these findings imply that statistical comparisons of performance across areas, or across subject groups require great care.

While our treatment is based on simulations that make simplifying assumptions (e.g. about the hemodynamic response function and spatial correlations), focuses on a support vector machine for classification, and investigates a two-class block-design experiment only, the issues are quite general and apply at least conceptually to any study that uses multi-voxel pattern classification. The goal of this report is not to discredit previous research, nor do we claim that we are the first to note these problems. Our goal is to analyze the problems, illustrate them with simulated data, alert the reader to the seriousness of the problems and the inadequacy of some purported solutions, and provide alternative solutions where available.

## Methods

All experiments used simulated data based on simple assumptions about the nature of neural signals and neurovascular coupling. For simplicity we only considered experimental paradigms with two classes (A and B).

Unless otherwise specified, the neural response in each voxel at each point in time was simulated as a random number drawn from a Gaussian distribution with zero mean and unity variance. This was so irrespective of whether the point in time corresponded to Class A or Class B. Thus, there was no relation between neural activity and the classes of the experimental design, nor was there any correlation among voxels. In other words, this simulated brain had no knowledge whatsoever of the experimental conditions. Using such a ‘null’ data set is a convenient way to test an analysis procedure as any significant classification performance must be artifactual and therefore a reflection of an inappropriate data analysis. Put differently, because there was no true class-related signal in the simulated neural data, every significant classification was a false positive.

From these simulated neural signals we derived BOLD responses per voxel by convolving the neural activity with a hemodynamic response function (HRF) [9]: *h*=(*t*)^a^ *e* ^-t/b^, with *a*=8.6 and *b*=0.547.

We simulated a canonical block-design experiment in which blocks of class A alternated with blocks of class B. The TR was 2s, and each block was 32 seconds long, hence it contained 16 volumes, which we refer to as time points. In different experiments, each block was repeated 5, 10, or 15 times, resulting in 160, 320, or 480 time points per experiment. We removed the first 8 time points (16s) from each block to avoid spill-over activation from the previous block.

## Results

We generated simulated data (See Methods) for a typical block-design experiment with two classes (A and B). The Results section is divided into three subsections, covering the typical analysis of such data. The first (Estimation) discusses the estimation of the classification performance based on such a data set. The second (Significance Testing) shows how standard methods to assess the statistical significance of classification performance can fail dramatically and provides an alternative, reliable test. The third (Performance Comparisons) analyzes issues that arise when one wishes to compare performance across areas or across subject groups.

### Estimation

We trained a linear support vector machine (SVM [10–12]:) on the training data (80% of the time points randomly chosen from the entire data set, while ensuring that each class occurred equally often in the set) and tested the classifier on the remaining 20% of the data. This random cross-validation (CV) was repeated 100 times per data set, with independent random assignments to training and test sets for each run. The performance was defined as the mean performance across these CV repeats.

**Figure 1:**
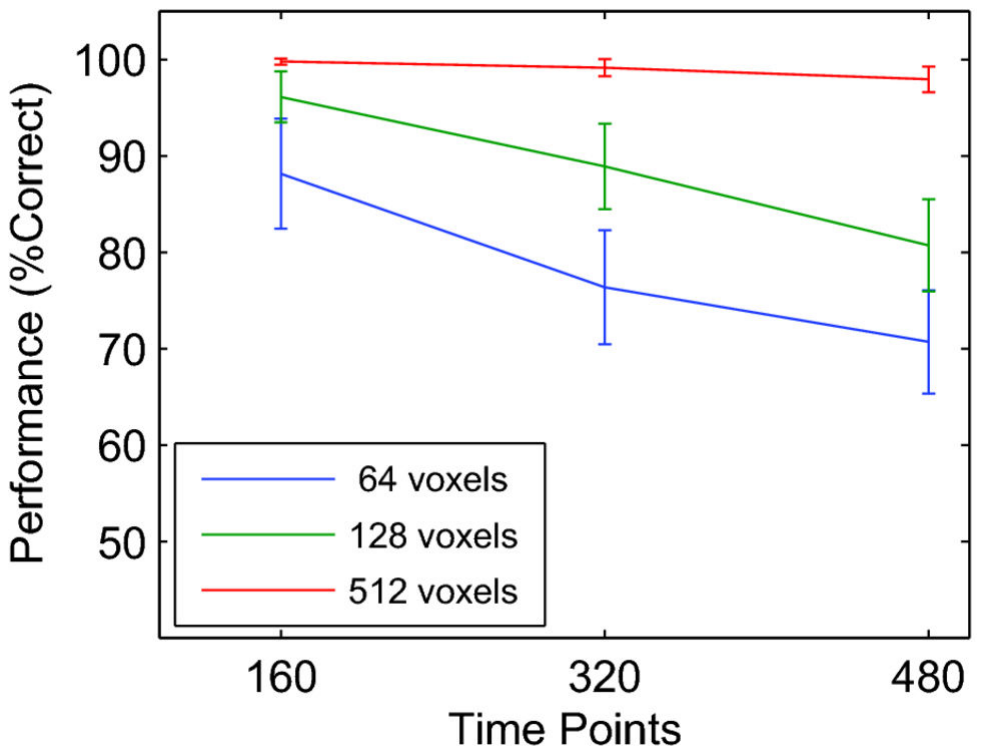
Mean classification performance in a two-class block design experiment. The randomly cross-validated percentage correct (y-axis) is shown as a function of the number of time points (x-axis) for three ROI sizes (64, 128,and 512 voxels). Error bars show the standard deviation across 1000 independent null data sets. This figure shows that using randomly chosen time points for cross-validation generates spurious classification performance.

Figure 1a shows classification performance as a function of the number of time points for regions of interest (ROIs) that contained different numbers of voxels. The performance was always well above “chance” (50% for this 2 class problem) and highest for a small number of time points and a large number of voxels. This is mysterious; how can a classifier perform above chance even though the underlying neural signal was pure noise and had no relationship with the classes?

The explanation of this above chance performance lies in the sluggishness of the hemodynamic response function. Even though the neural signals were independent across time points, the convolution with the slow HRF generated a BOLD response in which nearby time points were necessarily similar. Because nearby time points in a block design also typically correspond to the same class, there is in fact real “signal” that allows the classifier to perform above chance.

This problem is more pronounced for large ROIs because the BOLD response in each voxel contributes some independent (spurious) signal because the underlying neural signals were independently drawn from Gaussian distributions. The problem is most pronounced for a small number of time points because in a smaller data set, more test time points will be (temporally) near the training time points and, therefore more likely to share the spurious signal.

The solution to this particular problem is to leave enough time between training and test data such that the slow BOLD response cannot introduce temporal correlations between them. In practice, this is most easily done by training the classifier on one set of randomly chosen blocks (rather than randomly chosen time points), and testing it on a separate set of blocks. This is called Leave-Block-Out cross-validation (LBO-CV) and is used in many MVPA studies [4]. As long as the time between training and test blocks is longer than the expected duration of the hemodynamic response, this procedure should resolve this particular issue.

We re-analyzed the above experiment, now using LBO-CV. In each CV repeat we chose a single block from each class to serve as test set, and used the remaining blocks as training set. This was repeated such that each block served as test set once. The performance was averaged over these repeats.

**Figure 2:**
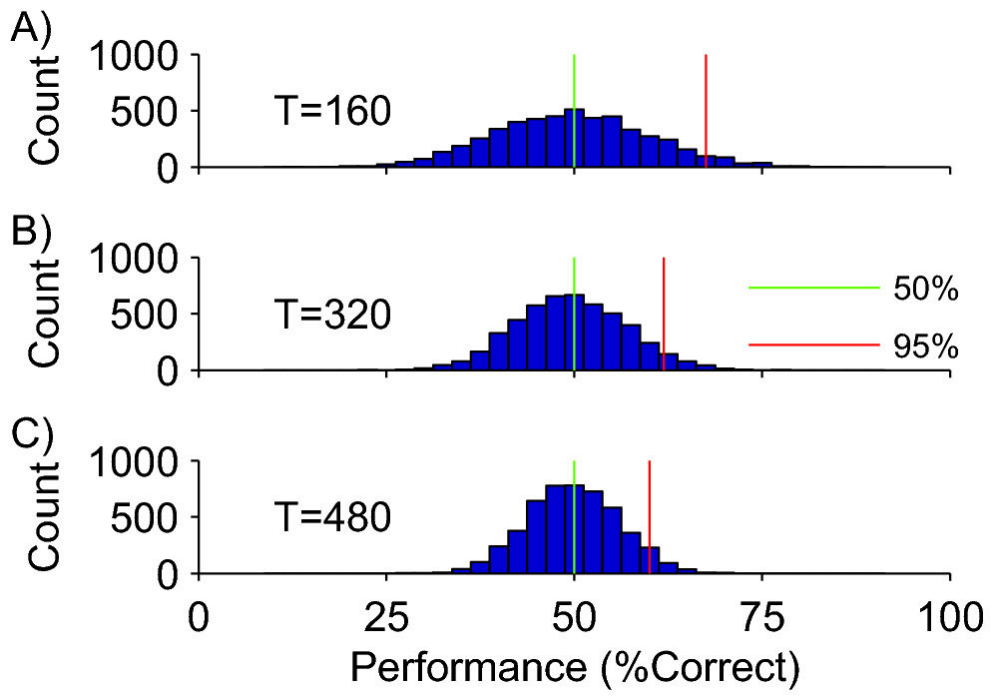
Leave-Block-Out cross-validation. Each panel shows the distribution of classification performance across 5000 independent null data sets containing 512 voxels. A) Experiments with 160 time points. B) Experiments with 320 time points. C) Experiments with 480 time points. The green line shows the median of the distribution, the red line the 95^th^ percentile. LBO-CV correctly estimated performance on average (50%), but the performance null distributions were very wide; even on null data, a performance in the 60-70% range is not unlikely.

Figure 2 shows the results. Each panel shows a histogram of average LBO-CV performance values for experiments with different numbers of time points and a fixed number of 512 voxels. The green lines show the median performance, which is indistinguishable from 50% in each case; this confirms our intuition that on average the SVM should not be able to do this classification task.

Now that we have a method (LBO-CV) that correctly estimates the average classification performance, we move on to the next problem; assessing the statistical significance of a particular performance value.

### Significance Testing

Figure 2 demonstrates that significance testing is not a trivial issue. Even though the average performance was 50% in each case, the classifier often had levels of performance that were considerably above 50%. This implies that obtaining a classification performance in, for instance, the 60-70% range is not unlikely even when the neural data consist entirely of Gaussian noise. Moreover, the variance of the performance distributions depended on the number of time points (compare across panels A-C in Figure 2). A valid statistical test must take this into account.

To assess the validity of statistical tests that have been used in some MVPA studies, we simulated 1000 null data sets (neural signals and classes). The simulation procedure was identical to that used for Figure 2. For each of these data sets, we performed a statistical test of the null hypothesis that there was no association between signals and classes at the α=0.05 level. Because there was no true signal in our simulations, every performance value that a test deemed significant was a false positive. For the alpha-level used, a valid statistical test would generate a positive result in 5% of cases. In the following sections, we show how commonly used statistical methods (binomial test, T-test) generate far more false positives. The common underlying reason is that these tests assume independence of performance estimates, even though the data that these estimates are based upon have considerable overlap. We then describe a permutation test that avoids this problem and correctly assesses significance.

#### Binomial Test

Because each of the N test sets in LBO-CV is independent, and one would expect 50% (“chance”) performance on each test set, one might reason that –under the null hypothesis-the LBO-CV procedure is equivalent to flipping a coin N times. If this argument were correct, one could determine significance with a simple binomial test. For the specific cross-validation procedure used in Figure 2, N was equal to the number of blocks because each block was used as test set once. Consider for instance the experiment in Figure 2c, which had 480 time points (15 repeated blocks of each condition). Using a binomial test with N =15 failed to reject the null hypothesis for any of the 1000 data sets. From this, one might conclude that the binomial test is overly conservative (5% of the tests should have rejected the null hypothesis), but the next example shows that this interpretation is incorrect.

**Figure 3:**
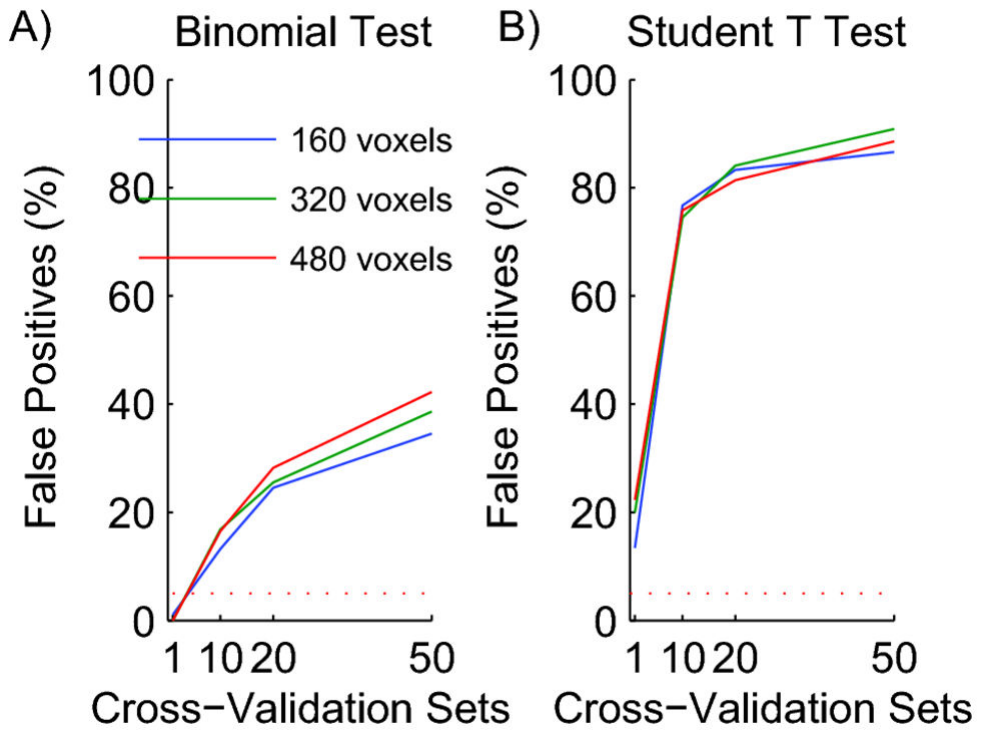
Traditional parametric tests are invalid for the assessment of statistical significance of classification performance. A) Binomial Test. B) Student’s T-test. Both panels show the percentage of 1000 experiments with null data that were considered significant at the 0.05 level. Because the data had no signal, all of these are false positives. The dashed lines show the expected percentage of false positives for a correct statistical test (5%). The use of multiple CV sets led to large overestimates of significance. This analysis shows that neither binomial, nor Student’s T-tests can be used to determine the statistical significance of classifier performance.

A good method to improve the estimate of the average classification performance is to test each block multiple times (each time in combination with a different block from another class). However, if the number of repeated CV sets is used in a binomial test, statistical significance is greatly overestimated. To continue the example based on the data of Figure 2c, we re-calculated the performance 10 times per block, each time with a different paired block from the other condition. The mean performance across the 10 sets was still 50%, but using the number of repetitions (N=10*15) in the binomial test led to the incorrect conclusion that 16% of the findings were statistically significant. Figure 3a shows how the percentage of false positives increased with the number of CV repeats.

The reason that the binomial test gives incorrect results is that –even in a single CV repeat-the data sets used to *train* the classifier always overlap. Moreover, when using multiple CV-repeats, even the data sets used to *test* the classifier will overlap. In other words, the multiple assessments of performance are not independent coin flips. Hence, regardless of whether one does single or multiple CV repeats, the binomial test in which separate CV tests are used as independent samples is invalid and does not provide an accurate assessment of the statistical significance of classification performance.

#### Student’s T-Test

Given its ubiquity one might be tempted to use a T-test to determine whether the mean performance value over the N test sets is different from 50%. We analyzed our null data with T-tests at alpha=0.05. Figure 3b shows the false positive rates. Even for a single LBO-CV repeat, they exceeded 5%. The main reason why the T-test fails is again the overlap between the training sets. As a consequence, the performance samples from these multiple CV sets are not independent, and a T-Test is not valid. The overlap between sets increases with the number of sets that are constructed from a fixed size data set, which explains why the fraction of false positive results increases with the number of CV repeats (Figure 3b).

#### Error bars

Often, MVPA classification performance values are presented with error bars corresponding to the standard error measured over CV sets. This data presentation suggests that the error bars allow one to assess the statistical significance by determining whether the distance between performance and chance level (50%) is large compared to the length of the error bar. However, because this eye ball test is implicitly based on a T-test, the simulations of Figure 3b show that such a representation is misleading.

In addition, we note that while chance performance is 50% on average, a particular data set drawn from Gaussian noise can easily generate cross-validated performance well above 50% (as shown in Figure 2). In other words, “chance” is not a single value, but a distribution of values. To allow eye-ball tests of significance one must provide error bars on the chance level in addition to error bars on the performance estimate. For instance, a useful convention would be to show the 95^th^ percentile of the performance null distribution (see below).

#### Permutation Tests

The parametric tests investigated above fail to provide an accurate assessment of statistical significance. A better approach uses a permutation test [7]. In such a test one creates null data sets by randomly shuffling the class labels; this destroys any potential relationship between classes and signals. Second, one analyzes the performance on this null data set in the same way as the original data. This process is repeated many times to create a distribution of the expected performance levels under the null hypothesis (i.e. that there is no relationship between signal and classes). The performance estimated on the true data is considered significant (at p<0.05) if it is larger than the 95^th^ percentile of the null distribution. This approach makes no assumptions about the shape of the performance distribution, or about the shape of the distribution of the raw signals. All of these distributions are estimated from the data. While computationally more demanding than parametric tests these permutation tests are well within the reach of modern day computers and has been advocated as an appropriate test for image based analysis [8].

As there are multiple ways to implement a permutation test it is important to discuss which one leads to correct assessments of significance. We discuss two approaches here.

### Global Permutation Test

**Figure 4:**
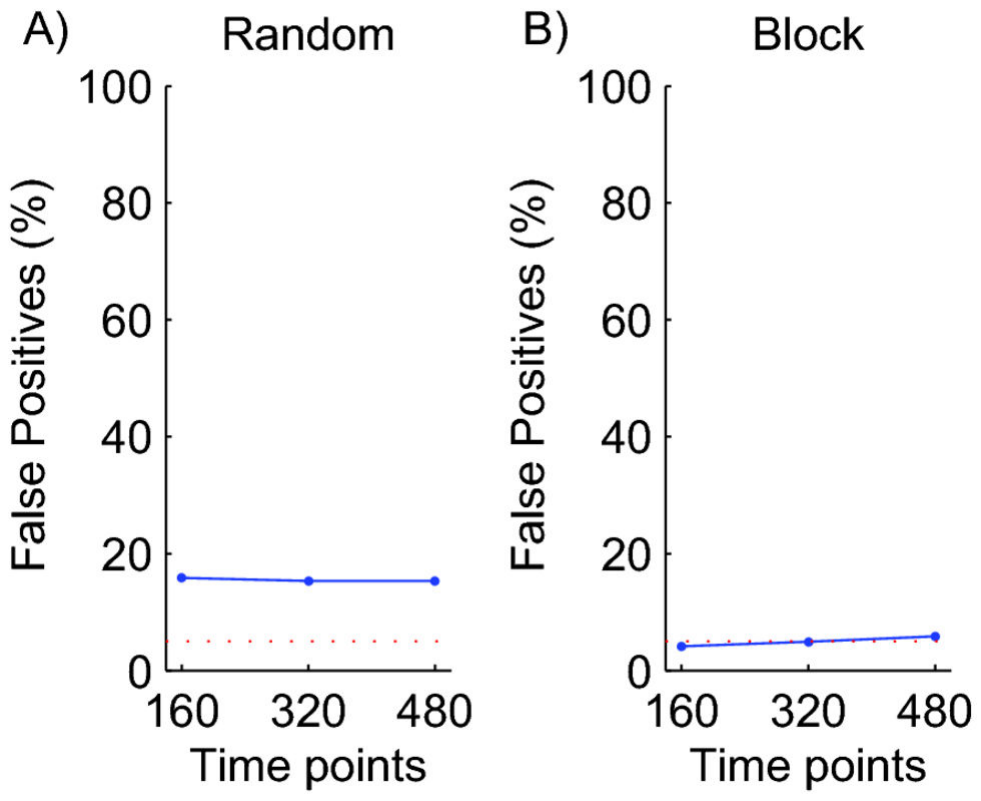
Permutation Tests. A) Global permutations. B) Block permutations. Each data point shows the percentage of experiments (out of 1000) based on null data that were considered significant according to a statistical test at the 0.05 level. The dashed lines show the expected percentage of false positives for a correct statistical test (5%). This figure shows that random, global permutations lead to overestimates of significance, while the block permutation test in B correctly estimates the significance of the classification performance.

In what we call a global permutation test, the relationship between BOLD response and classes is destroyed by randomly shuffling the labels across the whole data set. A label anywhere in the data set can be shuffled to a new time point anywhere else in the data set. Figure 4a shows the fraction of null data sets with a classification performance that was above the 95^th^ percentile of the cumulative distribution based on 1000 random global permutations of the class labels. Clearly there are too many false-positives; the test overestimates statistical significance.

The reason for this high false-positive rate is that in the original (non-shuffled) data set, nearby points in time had similar signals and similar class labels. Hence if time point n happened to be classified correctly, then it was likely that n-1 and n+1 would be classified correctly too. The converse is true as well; if n happened to be classified incorrectly, then n ± 1 would most probably be incorrect too. These temporal correlations increase the variance of the distribution of performance values when compared to a data set without temporal correlations. Due to this larger variance one is more likely to find performance values in the original (non-shuffled) data set that are above the 95^th^ or below the 5^th^ percentile of the shuffled data set, even though the average performance in both sets is 50%.

The underlying problem here is the same as that in performance estimation: the sluggishness of the BOLD response (See above). The solution to this problem is to keep the block structure of the experiment intact when shuffling the data.

### Block Permutation Test

Rather than shuffling single labels, one should shuffle entire blocks of labels. This allows the temporal correlations within the block to play the same role in the shuffled data set as they do in the non-shuffled data set. Second, to ensure that performance is measured equally on all classes, each of the permutations should have an equal number of blocks from each class. We refer to this procedure as a (balanced) block permutation test. Figure 4b shows that using this procedure, only 5% of all simulated data sets were considered to have a mean performance that was statistically significant at the 0.05 level. This is exactly what one would expect for null data. The block permutation test is the only test of the statistical significance of classifier performance that we can recommend.

### Performance comparisons

**Figure 5:**
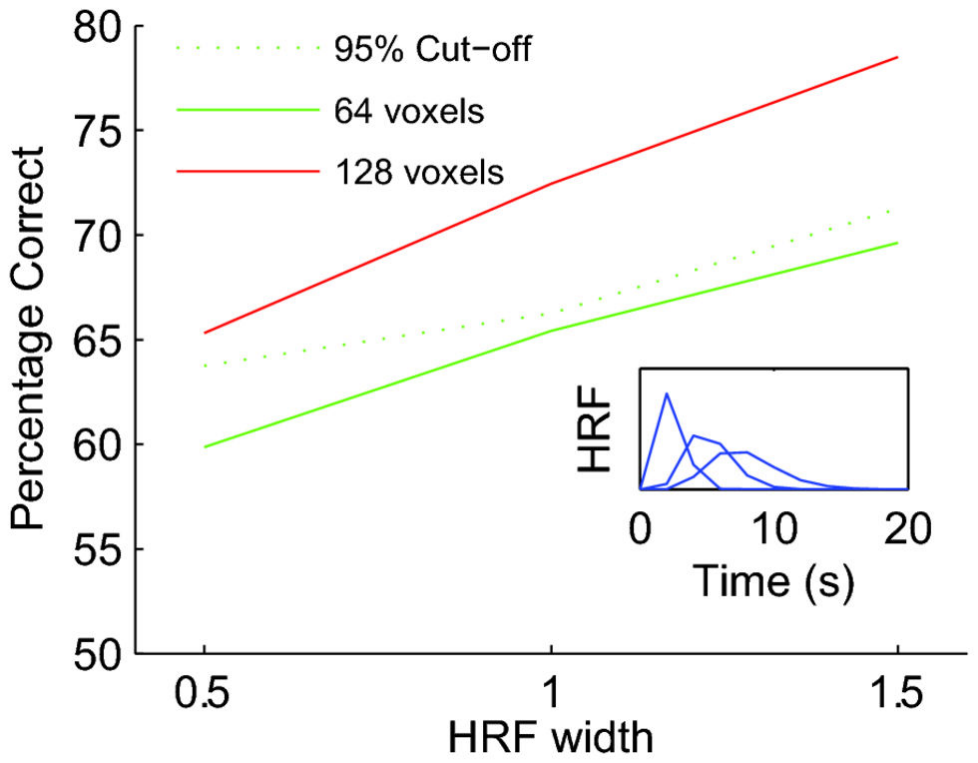
The HRF in a region of interest affects classification performance. The green and red curves show mean performance on data sets with true neural signal (signal strength s was 0.1; see main text), Green curve: mean performance on 64 voxels, red curve: mean performance on 128 voxels. Dashed curve: level of performance achieved on 95% of a null data sets consisting of 64 voxels (i.e. the significance cut-off). This figure shows that stretching the HRF by 50% can increase classification performance by nearly 10%. In other words, if it is possible that the HRF could differ between two data sets, then a difference in classification performance cannot be used to infer a difference in the underlying neural response.

As explained above, spurious classification performance in a block design experiment arises from the temporal correlations in the BOLD response induced by the slow hemodynamic response function. This suggests that performance measures will depend on the shape of the HRF. To investigate this, we modeled three HRFs by scaling their width by a factor of 0.5 (a short HRF), 1 (the canonical HRF) or 1.5 (a long HRF). The insert in Figure 5 shows the shape of these three HRFs.

We created data sets with a true class-related signal by injecting one neural activity pattern into blocks of class A and a different activity pattern into blocks of class B. We first simulated neural noise as we did in all previous simulations by drawing random numbers from a Gaussian distribution with zero mean and unit variance. Second, to introduce true signal, we drew one random number per voxel from a Gaussian distribution with a mean of zero and standard deviation *s*. This resulted in a single neural activity pattern across voxels, which we added to each of the time points corresponding to class A. Third we drew a new random pattern across voxels from the same distribution and added this to the time points corresponding to class B. When *s* is large, the difference in each voxel between activity in time points corresponding to A and those corresponding to B is large, hence *s* is a measure of signal.

Figure 5 shows the mean classification performance on such a data set as a function of the HRF width. The green curves shows a simulation based on 64 voxels, the red curve is based on 128 voxels. Both simulations used s=0.1. Clearly, performance estimates increased with HRF width, even though the true neural signal in each of these simulations was the same.

The dashed curve shows the 95^th^ percentile of the performance distribution of block-shuffled data (64 voxels). In other words, it is the cut-off percentage above which the block permutation test will label performance as significant. Clearly the cut-off also increased with the duration of the HRF. Importantly, the green solid curve is below the dashed curve for all HRFs. This means that statements about the statistical significance of performance in a single region were not strongly affected by the HRF width (i.e. even if we do not know the HRF, the block-permutation test will correctly assess the significance of the performance).

This same analysis, however, also shows that the numerical value of the performance is not a meaningful quantification of the information present in the neural activity of an area. First of all, it is clear that the number of voxels in an area strongly affects the performance. The signal per voxel in the larger area (red curve) was the same as that in the smaller area (green curve), nevertheless the larger area always outperformed the smaller area. This is expected as the signal in each voxel is an independent source of information (in these simulations; in the brain spatial correlations likely exist; see below). Second, the figure also shows that an area with the same number of voxels, and the same signal per voxel, but wider HRF performed better on the classification task. For this particular simulation, stretching the HRF by 50% increased the performance nearly 10%.

This analysis shows that when two regions of interest lead to different classification performances, one cannot infer that the neural representations in those areas have different neural signals. The difference could be due to differences in the size of the areas (which in principle is easy to control for, but see below) or differences in the HRF. The severity of this problem will rely on detailed properties of the HRF, as well as signal to noise ratios.

#### Spatial Correlations

**Figure 6:**
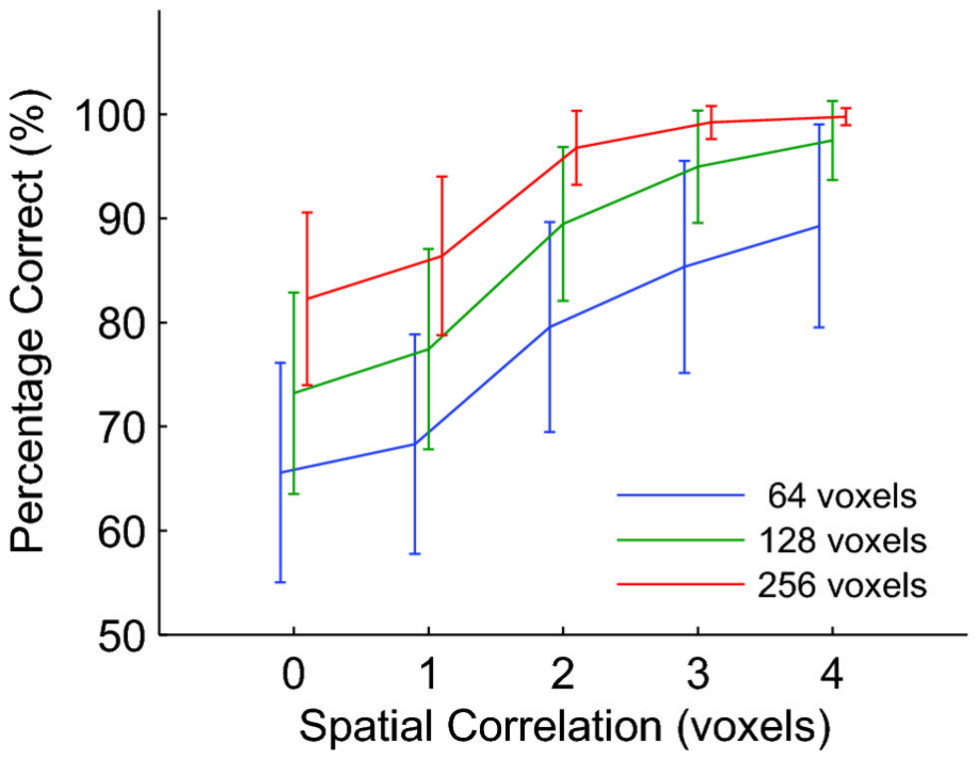
Spatial correlations affect classification performance. Each curve shows the mean classification performance based on a data sets containing true signal (s = 0.1) for a different ROI size as shown in the legend. Error bars show ±1 standard deviation across 1000 independent data sets. Spatial correlation on the x-axis represents the standard deviation of the Gaussian spatial filter, which we use to simulate the spatial spread of the BOLD response. Data points are offset horizontally for visual clarity. This figure shows that spatial correlations in the BOLD response induced by a spatially unspecific vasculature can inflate classification performance estimates.

In all simulations up to this point, we assumed that each voxel contained an independent neural signal. In a typical fMRI experiment, however, the signals in neighboring voxels are correlated. In this section we investigate how spatial correlations affect MVPA. We again introduced a pattern to all class A time points and a different pattern to all class B time points (as in Figure 5), but now created spatial correlations in the signal by convolving each pattern with a Gaussian filter. This could be viewed as a crude model of the situation where neurons in one voxel carry class-related signals, but because the neighboring voxels use some of the same blood supply their BOLD response also contains some signal. Figure 6 shows that performance increased sigmoidally with the spread of correlations as measured by the width of the Gaussian filter.

These simulations show that an area in which the vasculature leads to spatially widespread changes in blood oxygenation could generate better MVPA performance than an area in which the vasculature targets very specific regions, even if the underlying neural activity in the two areas is identical. This is another reason why a comparison of performance between areas – even when the number of voxels is equal – can be problematic.

In some studies, performance comparisons across areas of unequal size are done by selecting a subset of voxels from the larger area that match the total number of voxels in the small area [13]. Our analysis suggests that this selection preprocessing should be done with care as a random selection of voxels from a large area is likely to be less correlated than neighboring voxels in a small area. These differences in correlations could lead to differences in classification performance without any differences in the underlying neural signals.

Finally, we note that the variance of the performance null distributions also depends on the number of time points (Figure 2), and the number of voxels (not shown). Therefore non-parametric tests are better comparisons of classification performance on data that differ along those dimensions (whether by preprocessing or experimental design) than tests that assume homoscedasticity (e.g. ANOVA).

## Discussion

We simulated BOLD imaging experiments and analyzed them with multi-voxel pattern analysis. The temporal correlations induced by the hemodynamic response function led to the well-known issue of performance overestimation, which was resolved with leave-block-out cross-validation. We also identified another important, and underappreciated problem in MVPA; the assessment of statistical significance, which we argue should only be done using block-level permutation tests and not with binomial or T-tests. Finally, we show that temporal and spatial correlations (such as those induced by the HRF) prevent a meaningful direct comparison of classification performances between areas, or subject groups in which those HRFs could be different.

### Generality

Our simulations made various assumptions and an important consideration for the practical application of our results is how general they are. For instance, we chose a specific classifier (the support vector machine), a specific pattern (the raw BOLD activity across voxels), a gamma-shaped hemodynamic response function, and a two-class block-design.

An alternative to the support vector machine is the use of the Pearson correlation for classification. In this approach, one calculates the Pearson correlation of a test pattern with the training patterns from each class, and classifies the test as belonging to the class with the highest Pearson correlation. We performed all simulations shown here with this classifier (not shown), and the results are qualitatively the same. This should not be surprising as the essence of the problem is not the classifier but the spatiotemporal correlations in the BOLD signal and the overlap of training sets in repeated cross-validation.

In our simulations the patterns simply represented the BOLD activity across voxels. Some studies, however, use more complex multivariate measures of brain activity. For instance, the pattern of beta weights resulting from a GLM analysis. We see no reason to believe that MVPA based on such patterns would be immune to the estimation problems highlighted in this paper given that temporal correlations can at least in principle affect beta weights. Similarly, because the issues surrounding significance testing arise from the overlap between training and test sets, and not from the nature of the patterns themselves, the same concerns apply. Moreover, with these kinds of approaches, the potential influence of an incorrect assumption about the HRF enters at an earlier stage of analysis as it could amplify or destroy a pattern that is present in the raw data. However, as the devil is in the details, careful simulations of combined GLM & MVPA analyses may be needed to understand the quantitative extent to which this approach suffers from the problems discussed in this paper.

We used a simplified formula to simulate the hemodynamic response (See methods), and this may not accurately reflect the real HRF. But here too, the details do not affect the qualitative outcome (simulations using a simple boxcar HRF led to qualitatively similar results; not shown). Any reasonable assumption about the HRF will introduce temporal correlations on a time scale of several seconds and these correlations lead to the potential problems we highlight in this paper.

While we considered only simple ABABAB block designs, the importance of using LBO-CV applies to many experimental designs. For instance they apply to multi-class designs (ABCABCABC), because those are typically based on solving multiple pair wise classification problems, each of which will suffer from the problems illustrated here. But they also apply to fast event-related designs where careful balancing of the sequence of events from different classes is required to avoid spurious performance based on temporal order effects. Only in a design where the BOLD data used by the classifier are known to be independent can one use random cross-validation, and a global permutation test for significance. This may apply to some slow event-related designs where the time between measurements is longer than the duration of the HRF. In a typical MVPA analysis based on folded cross-validation testing, however, true independence is unlikely. Moreover, we note that there is ample opportunity in the brain for temporal correlations on time scales even longer than the HRF – such as those associated with breathing, head motion, or scanner drift. These possible confounds require careful attention in future work.

The issues of estimation, significance testing, and performance comparison apply quite universally to most if not all multi-voxel classification problems. However, this does not mean that they affect each of those approaches, or even each experiment using the same approach, in quantitatively similar ways. In other words, the issues are potentially present, our simulations show that they can be large, but the true size of the effects has to be investigated separately for each data set. Most importantly, simulations like the ones we performed here cannot be used as a proxy for a statistical test. For instance, an experimenter with a 480 time point, two-class experiment and 69% classification performance cannot point to our Figure 2C and claim that their result is statistically significant. Instead, this researcher should use LBO-CV, carefully consider all the possible dependencies of training and tests sets, and perform a block permutation test to assess significance.

### Group level analysis

If one is willing to forego an assessment of significance at the single subject level, some of the issues discussed here can be avoided. For instance, as long as an unbiased estimate of performance has been obtained in each subject (i.e. using LBO-CV) significance can be tested using a T-test to compare the mean performance across subjects with chance performance. We note, however, that this does not resolve the problems associated with the comparison between areas or subject groups; inferences about differences in neural representations are only valid to the extent that the HRF can be assumed (or shown) to be the same.

### Performance Comparisons

Considerable additional complexities arise when one considers the presence of true class-related signals; surely the situation of most interest in experimental research. Our simulations (Figure 5 and Figure 6) show that a difference in performance between two regions of interest does not guarantee a difference in the underlying neural signals; it could be due to differences in neurovascular coupling that change temporal (Figure 5) or spatial (Figure 6) correlations. This could for instance affect comparisons of performance between age groups, or healthy and diseased brains. A possible solution to this problem is to measure the HRF or the spatial correlations separately in the areas or subject groups and explicitly incorporate this into the analysis. This could help to understand whether classification performance differences should be attributed to vascular or neural effects. Of course these particular considerations also apply to standard analyses based on single-voxel activation, as pointed out by Logothetis and Wandell [14].

A comparison between an area that classifies significantly and one that does not is often made implicitly. Such a comparison requires a statistical test that directly tests the null hypothesis of no difference between the two areas [15]. But, even when that statistical comparison is done correctly, the interpretation of a performance difference is complicated by the possible influence of the vasculature. In this context it is also worth pointing out that little is known about false negatives in MVPA, i.e. true class-related neural signals that are not detected by MVPA. Investigating these quantitatively using a simulation approach is difficult as it requires realistic models of neural signals and the HRF.

In Figure 6, we show that signal correlations increase performance. It is important to note that noise correlations can undo some or all of this, hence final classification performance will depend in detail on the relative strength (and sign) of noise and signal correlations. Averbeck et al [16] review these issues in the context of neural population decoding and provide an intuitive geometrical interpretation of why signal correlations generate higher classifier performance. Our simulated spatial correlations were only intended to illustrate a conceptual point, using simplistic assumptions about the vasculature. For a more detailed treatment that focuses on the possible linking between vasculature and classification, we refer to Gardner [17] and Kriegeskorte et al [18].

## Conclusion

Multi-voxel pattern classification analysis is a powerful tool to determine whether the BOLD response in a set of brain voxels contains information that is useful for a particular behavioral task. The real question of most interest, however, is whether the neural signals that generate these BOLD responses contain information. Assessing this is complicated by the temporal and spatial correlations induced by the hemodynamic response function, and the overlap among cross-validation training and test sets. Binomial and Student T tests are inappropriate tests of significance, and we argue that only a block-level permutation test should be used. Comparisons of performance between regions or subjects should be treated with caution as any difference in spatial or temporal correlations, such as those introduced by differences in hemodynamic coupling, can increase classification performance without any change in the underlying neural signals.
